# Adipose-derived mesenchymal stem cells modulate CD14^++^CD16^+^ expression on monocytes from sepsis patients in vitro via prostaglandin E2

**DOI:** 10.1186/s13287-017-0546-x

**Published:** 2017-04-26

**Authors:** Guanguan Qiu, Guoping Zheng, Menghua Ge, Lanfang Huang, Haijiang Tong, Ping Chen, Dengming Lai, Yaoqin Hu, Baoli Cheng, Qiang Shu, Jianguo Xu

**Affiliations:** 1 Shaoxing Second Hospital, 123 Yanan Road, Shaoxing, Zhejiang 312000 China; 2 The Children’s Hospital of Zhejiang University School of Medicine, 3333 Binsheng Road, Hangzhou, Zhejiang 310051 China; 3 The First Affiliated Hospital of Zhejiang University School of Medicine, 79 Qingchun Road, Hanghzou, Zhejiang 310003 China

**Keywords:** Adipose-derived mesenchymal stem (stromal) cells, CD14^++^CD16^+^, Monocytes, COX-2, PGE2, Sepsis

## Abstract

**Background:**

Mesenchymal stem cells (MSCs) have been shown to reduce sepsis-induced inflammation and improve survival in mouse models of sepsis. CD16^+^ monocytes are proinflammatory and abundant in inflammatory conditions such as sepsis. The primary objective in this exploratory study was to determine the effects of adipose-derived MSCs (ASCs) on three subsets of monocytes from sepsis patients in vitro and to delineate the underlying mechanism.

**Methods:**

This is a prospective cohort study of patients admitted to the medical intensive care unit (ICU) at an academic medical center. The levels of CD14^++^CD16^+^, CD14^+^CD16^++^, and CD14^++^CD16^–^ monocytes from 23 patients in the early phase of severe sepsis or septic shock as well as 25 healthy volunteers were determined via flow cytometry after coculture with or without ASCs. To determine the molecular mechanisms, the effects of exogenous prostaglandin E2 (PGE2) and the cyclooxygenase-2 (COX-2) inhibitor NS-398 on monocyte phenotypes and cytokine expression were also examined.

**Results:**

Basal levels of CD14^++^CD16^+^ but not CD14^+^CD16^++^ monocytes were significantly elevated in severe sepsis and septic shock. A positive linear relationship existed between the levels of CD14^++^CD16^+^ monocytes and the Acute Physiology and Chronic Health Evaluation (APACHE) II score as well as Sequential Organ Failure Assessment (SOFA) score. Coculture of ASCs with monocytes from sepsis patients for 24 h significantly reduced CD14^++^CD16^+^ expression while increasing the CD14^++^CD16^–^ phenotype. The coculture also significantly elevated PGE2, COX-2, and prostaglandin E2 receptor (EP)4 levels generated from monocytes. Functionally, ASCs reduced the tumor necrosis factor (TNF)-α and increased the interleukin (IL)-10 secretion in monocytes of septic patients. Furthermore, the effects of ASCs on the CD14^++^CD16^+^ phenotype and cytokine expression were mimicked by exogenous PGE2 and abolished by the COX-2 inhibitor NS-398. Additionally, ASCs also modified levels of monocyte phenotypes in a mouse model of sepsis.

**Conclusions:**

Levels of CD14^++^CD16^+^ monocytes positively correlate with disease severity scores in the early phase of severe sepsis and septic shock. ASCs switch monocytes of sepsis patients from CD14^++^CD16^+^ to CD14^++^CD16^–^ in vitro and modulate the production of inflammatory cytokines. The immunomodulatory effect of ASCs on monocytes is PGE2-dependent. ASCs may exert their therapeutic effect on sepsis via altering monocyte phenotypes and functions.

**Electronic supplementary material:**

The online version of this article (doi:10.1186/s13287-017-0546-x) contains supplementary material, which is available to authorized users.

## Background

Sepsis is a major clinical problem and the leading cause of death in intensive care units (ICUs). In the US, there are over 750,000 new cases and over 200,000 deaths annually [1]. In the early phase of sepsis, the disease is characterized by a systemic inflammatory response syndrome with overproduction of proinflammatory mediators from neutrophils and monocytes/macrophages. These destructive inflammatory responses lead to multiorgan dysfunction [2]. There is a need for new therapeutic strategies to improve the outcome of sepsis.

Over the past decade, mesenchymal stem (stromal) cells (MSCs) have received wide attention as a novel treatment for many diseases. MSCs have been proven effective to treat diseases such as graft-versus-host diseases [3] and myocardial infarction [4]. Furthermore, Wilson et al. [5] and our group [6] have tested the clinical use of MSCs for acute respiratory distress syndrome (ARDS). Results from other groups [7] and our team [8] have previously shown that MSCs can ameliorate experimental lipopolysaccharide (LPS)-induced acute lung injury. MSCs have also been documented to reduce inflammation, enhance bacterial clearance, and improve survival in sepsis [9]. Mechanistically, MSCs exert their protective effects via the secretion of multiple paracrine factors capable of modulating the immune response. These soluble factors include indoleamine 2,3-dioxygenase (IDO), prostaglandin E2 (PGE2), transforming growth factor (TGF)-β, interleukin (IL)-10, IL-1 receptor antagonist, hepatocyte growth factor (HGF), tumor necrosis factor (TNF)-α stimulated gene 6 (TSG-6), and nitric oxide [10]. Recently, MSC-derived microvesicles were reported to ameliorate the lung injury via the expression of keratinocyte growth factor mRNA [11].

Nemeth at al. reported that MSCs attenuate sepsis in a mouse model via the PGE2 pathway by reprogramming host macrophages to increase their IL-10 production [12]. It has also been documented that human MSCs reduce mortality and bacteremia in Gram-negative sepsis in mice by enhancing the phagocytic activity of blood monocytes [13]. Monocytes are a heterogeneous population of cells. Based on the differential expression of CD14 (LPS receptor) and CD16 (FcγIII receptor), monocytes have been classified into three subsets: classical CD14^++^CD16^–^, intermediate CD14^++^CD16^+^, and nonclassical CD14^+^CD16^++^ [14]. The latter two subsets are summarized as CD16^+^ monocytes in earlier studies. CD16^+^ monocytes have been considered as proinflammatory and characterized by higher expression of proinflammatory cytokines and higher potency in antigen presentation [15]. This population occupies approximately 10% of monocytes in healthy adults and they are abundant in inflammatory conditions such as sepsis [16], rheumatoid arthritis [17], and HIV [18].

MSCs have been shown to affect the phenotype and function of monocytes. It has been documented that umbilical cord-derived MSCs suppress T-cell proliferation via PGE2-mediated monocyte function [19]. MSCs can also modulate the effect of monocytes on T-cell proliferation via HGF, which induces IL-10 production in monocytes through the ERK1/2 pathway [20]. In another study, MSCs have been reported to transform CD8^+^ T cells into a suppressive phenotype by inducing tolerogenic monocytes [21]. In addition, MSCs support follicular lymphoma cell growth through formation of proangiogenic monocytes [22]. Furthermore, MSCs alleviate sepsis in mice by enhancing the phagocytic activity of blood monocytes [13]. Finally, bone marrow-derived MSCs are able to induce monocyte emigration into the blood stream in response to LPS [23]. As evidenced by these data, monocytes are one of the first target cells for MSCs.

We hypothesized that adipose-derived MSCs (ASCs) could modulate monocyte subsets in sepsis patients via the PGE2 pathway. The levels of CD14^++^CD16^+^, CD14^+^CD16^++^, and CD14^++^CD16^–^ monocytes in the early phase of severe sepsis and septic shock were determined with or without coculture of ASCs. To study the mechanisms of phenotype modulation, PGE2, cyclooxygenase-2 (COX-2), and prostaglandin E2 receptor (EP)1–4 levels were examined in the cocultured cells. Furthermore, the levels of monocyte subsets from severe sepsis and septic shock patients were investigated in the presence of exogenous PGE2 or a COX-2-specific inhibitor. To study whether ASCs modulate monocyte function via PGE2, TNF-α and IL-10 levels were determined in the presence of exogenous PGE2 or a COX-2-specific inhibitor. Finally, the effect of ASCs on the distribution of monocytes was studied in vivo in a mouse model of sepsis.

## Methods

### Study subjects

Twenty-three patients in the early phase of severe sepsis or septic shock hospitalized in the ICU of Shaoxing Second Hospital were consecutively enrolled in the study between January 2015 and November 2015. Sepsis was defined according to the definition of the American College of Chest Physicians and Society of Critical Care Medicine Consensus Conference Committee [24]. Eligible patients were at least 18 years of age and diagnosed with severe sepsis or septic shock within the previous 24 h at any time during their stay in the ICU. Exclusion criteria included malignancy, chronic inflammatory diseases, traumatic brain injury, HBV/HCV infection, HIV infection, and refusal of consent. Twenty-five healthy volunteers served as controls. Informed consent was obtained from all study subjects. The study was performed in adherence with the Declaration of Helsinki and was approved by the Research Ethics Committee at Shaoxing Second Hospital (reference number 2014RL052).

### Human monocyte separation, culture, and analysis

Percoll density gradient followed by plastic adherence was used for human monocyte separation. Nine milliliters of heparinized peripheral blood from individual septic patients and healthy volunteers was mixed with 9 ml phosphate-buffered saline (PBS). Aliquots of the mixture (6 ml) were overlaid onto 6 ml Ficoll-Paque density gradient (Cedarlane, Burlington, NC, USA) in 15 ml tubes, which were then centrifuged at 2000 rpm for 20 min. Peripheral blood mononuclear cells (PBMC) at the interface were collected and washed twice with PBS. The PBMC were plated in six-well tissue culture plates (Life technologies, Grand Island, NY, USA) at 1 × 10^6^ cells/well in RPMI 1640 medium supplemented with 10% fetal bovine serum (Life technologies). After culture for 2 h at 37°C with 5% CO_2_ in the tissue culture incubator, nonadherent cells were removed. Adherent cells were cultured in RPMI 1640 medium for 24 h. This purification method resulted in monocytes with a purity of >90%. Subsets of CD14^++^CD16^–^, CD14^++^CD16^+^, and CD14^+^CD16^++^ monocytes were determined according to the surface expression pattern of CD14 and CD16. Cells were stained with PE anti-human CD14 (Catalog 325606; Biolegend, San Diego, CA, USA) and APC anti-human CD16 (Catalog 302012; Biolegend). Isotype-matched control antibodies were used to determine the cut-off between negative and positive populations. To differentiate the three subsets, an isotype control separated between CD14^++^CD16^–^ and CD14^++^CD16^+^. Then, two straight lines were extrapolated at both ends of the CD14^++^CD16^–^ to gate the intermediate CD14^++^CD16^+^ from the nonclassical CD14^+^CD16^++^ [25]. Cells were assayed by flow cytometry using the Cytomics FC 500MPL cytometer (Beckman Coulter, Brea, CA, USA), and data were analyzed using CXP Version 2.2 software (Beckman Coulter).

### ASC expansion

Normal human adipose-derived ASCs were purchased from ATCC (Catalog PCS-500-011; LOT 59753760, passage 2; Manassas, VA, USA). Characterization of the human ASCs was previously reported by our group [6]. Cells were resuspended in expansion media containing Dulbecco’s modified Eagle’s medium-low glucose supplemented with penicillin and streptomycin and 2% fetal bovine serum plus epidermal growth factor (EGF) and fibroblast growth factor (FGF; R&D Systems, Minneapolis, MN, USA) at a density of 4000 cells/cm^2^. Cultures were maintained at 37°C in a humidified atmosphere containing 5% CO_2_ in 100-mm dishes. When the cultures reached near confluence (>80%), the cells were detached by treatment with trypsin/EDTA and replated at a density of 4000 cells/cm^2^. ASCs between passages 5 and 6 were utilized for the study.

### Monocyte-ASC coculture

ASCs were plated into monoculture wells, Transwell inserts, and monocyte-ASC direct coculture wells overnight at a concentration of 250,000 cells/well in six-well plates in RPMI 1640 medium supplemented with 10% fetal bovine serum in a 5% CO_2_ incubator at 37°C. A total of 1 × 10^6^ freshly separated PBMC were then plated into monoculture wells, wells beneath Transwell inserts containing seeded ASCs, and direct coculture wells (4:1 PBMC/ASC ratio). After culture for 2 h, monocytes were adherent to the plates. The nonadherent PBMC were discarded by washing with PBS twice. ASCs and adherent monocytes were cultured for another 24 h with fresh RPMI 1640 medium supplemented with 10% fetal bovine serum. COX-2 and EP1–4 protein expression in mono- and cocultured cells was analyzed by Western blot analysis. Culture supernatants were harvested for PGE2 measurement. To investigate whether ASCs exerted their regulatory effect by PGE2 production, PGE2 (Sigma-Aldrich) or the PGE2 synthesis inhibitor NS-398 (Cayman Chemical, Ann Arbor, MI, USA) was added to the monocytes or coculture and cultured for 24 h.

### PGE2 and cytokine measurement

Cell culture supernatants were harvested and stored at –80 °C. Commercial enzyme-linked immunosorbent assay (ELISA) kits were used to determine TNF-α (eBioscience), IL-6 (eBioscience), IL-1β (eBioscience), IL-10 (R&D Systems), and PGE2 (R&D Systems) levels in the supernatants according to the manufacturers’ instructions.

### Western blot

Cells were harvested and lysed in lysis buffer (10 mM Tris-HCl, pH 7.4, 150 mM NaCl, 0.5% Nonidet P (NP)-40, 1 mM EDTA, 1 mM Na_3_VO_4_, and 1 mM PMSF). The protein concentration was determined using a BCA protein assay kit (Thermo Scientific, Hudson, NH, USA). Protein extracts (10 μg) were separated on sodium dodecyl sulfate-polyacrylamide gel electrophoresis (SDS-PAGE) and transferred to polyvinylidenefluoride membranes (Millipore, Billerica, MA, USA). Membranes were blocked with a “sealed liquid” (5% nonfat dry milk in 1 × TBS) for 1 h at room temperature. Then, membranes were incubated with a monoclonal human COX-2 antibody (Catalog MAB4198; R&D Systems) or EP1–4 antibodies (Cayman Chemical) at 4°C overnight. The blots were then washed three times with TBST buffer (150 mM NaCl, 10 mM Tris-HCl, pH 7.4, 0.1% Tween 20) and incubated for 1 h at room temperature with a horseradish peroxidase-conjugated secondary antibody (Catalog 70-GAM0072; MultiSciences). Finally, the blots were washed three more times with TBST and visualized via enzyme-linked chemiluminescence using the EZ-ECL kit (Biological Industries, Kibbutz Beit-Haemek, Israel).

### Mouse in vivo experiment and analysis

C57BL/6 mice at 8 weeks of age were inoculated intraperitoneally with 5 mg/kg LPS or PBS (as a control). Human ASCs were washed with warm PBS and resuspended at a concentration of 2 × 10^6^ cells per 0.2 ml PBS. Immediately after PBS or LPS treatment, 2 × 10^6^ ASCs or PBS (0.2 ml) were injected via the tail veins of the mice. Whole blood was collected via cardiac puncture into EDTA-containing tubes 24 h later. The blood was then treated with red blood cell lysis buffer (0.83% NH_4_Cl) for 5 min at room temperature and washed with PBS. Then, cell suspensions were washed with FACS buffer (PBS/1% FCS) and incubated with combinations of the following antibodies: PE anti-mouse F4/80 (eBioscience), APC anti-mouse CD11b (eBioscience), and PE-Cy7 anti-mouse Ly6C (eBioscience). Isotype-matched control antibodies were used to determine negative and positive cells.

### Statistical analysis

Data were summarized as mean ± standard error of the mean (SEM) for normal distribution, and median (interquartile range (IQR)) for skewed distribution. Correlations were analyzed by the Spearman’s correlation coefficient. Continuous variables between two groups with skewed distribution were compared by Mann–Whitney *U* test. Differences among multiple groups were analyzed for significance using the analysis of variance (ANOVA) test, followed by Dunn post hoc multiple comparisons. Differences were deemed statistically significant at *p* < 0.05. Data management and statistical analysis were performed using Graphpad Software (Prism 5.01, Graphpad Software).

## Results

### Study patient characteristics

Twenty-three patients with severe sepsis or septic shock were included in the study. Thirty-eight patients were excluded from the study based on the exclusion criteria. The demographic and clinical characteristics of the study patients are shown in Table 1. The major etiologies of sepsis were pneumonia (*n* = 16), burn wound infection (*n* = 2), and peritonitis (*n* = 2) (Table 1). Positive culture of microorganisms was reported in eighteen patients with 11 Gram-positive and 7 Gram-negative infections. Acute Physiology and Chronic Health Evaluation (APACHE) II and Sequential Organ Failure Assessment (SOFA) scores were determined at sepsis diagnosis. Nine patients did not survive sepsis.

**Table 1:** Demographic and clinical characteristics of sepsis patients

	Sepsis (*n* = 23)
Age (years)	66.2 ± 12.6
Male/female	14/9 (60.9%/39.1%)
SOFA score	11.3 ± 3.3
APACHE II score	22.0 ± 7.1
Death, *n*	9
Microbiology of patients (*n*)	
Gram-positive	11
Gram-negative	7
No organism cultured	5
Viral	0
Type of infection (*n*)	
Pneumonia	16
Peritonitis	2
Severe pancreatitis	1
Severe cholangitis	1
Cholecystitis	1
Burn wound infection	2 (5 and 7 days after ICU stay, respectively)

Data are presented as patient number or mean ± SD

*APACHE II* Acute Physiology and Chronic Evaluation II, *ICU* intensive car unit, *SOFA* Sequential Organ Failure Assessment

### Phenotype characteristics of ASCs

**Fig. 1:**
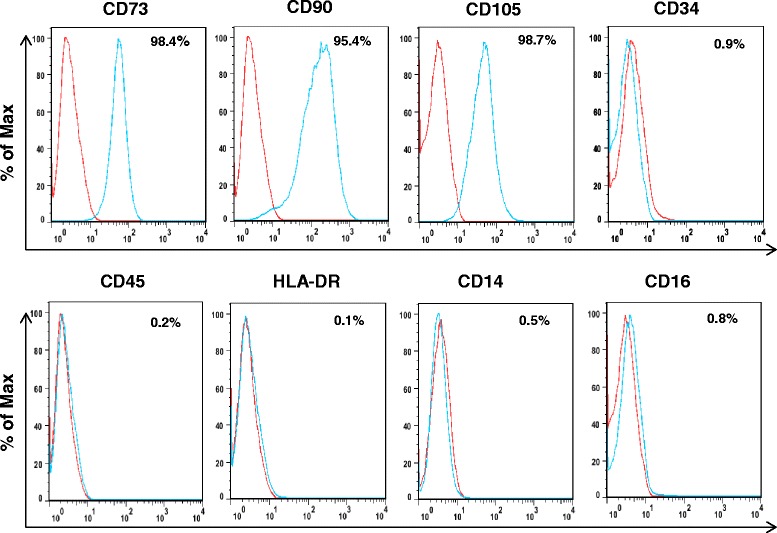
Phenotypic validation of the ASCs. Human ASCs at passage 6 were expanded in culture media. Flow cytometric analysis of surface markers showed ASCs to positively express CD105, CD73, and CD90 (*green*), but not HLA-DR, CD34, CD45, CD14, and CD16 compared with isotype controls (*red*)

The human ASCs for the study have been previously characterized by our group [6]. To ensure there is no drift in phenotype and no expression of CD14 and CD16 which are established markers for monocytes, surface protein expression at the end of expansion was determined by flow cytometry. The ASCs were positive for CD73 (98.4%), CD90 (95.4%), and CD105 (98.7%), but were negative for CD34 (0.9%), CD45 (0.2%), HLA-DR (0.1%), CD14 (0.5%), and CD16 (0.8%) (Fig. 1). Therefore, ASCs do not express CD14 and CD16 markers for monocytes.

### Levels of CD14^++^CD16^+^ monocytes are elevated in severe sepsis and septic shock

There were three subsets of cells, including CD14^++^CD16^–^, CD14^+^CD16^++^, and CD14^++^CD16^+^, in the cultured monocytes based on the surface expression of CD14 and CD16.

**Fig. 2:**
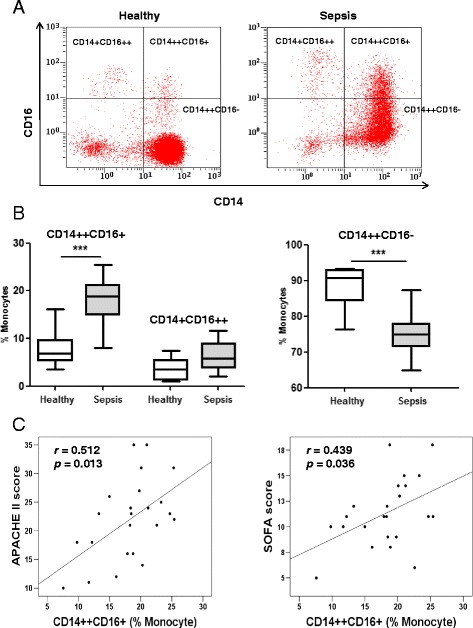
Levels of CD14^++^CD16^+^ monocytes in sepsis patients and their correlation with disease severity scores. **a** A representative flow cytometry plot showed the distribution of CD14^++^CD16^–^, CD14^++^CD16^+^ and CD14^+^CD16^++^ monocytes in healthy donors and sepsis patients. **b** The box and whisker plots represent the percentage of CD14^++^CD16^+^, CD14^+^CD16^++^, and CD14^++^CD16^–^ monocytes in the total monocyte population of healthy donors (*n* = 25) and sepsis patients (*n* = 23). The plot shows a median (*lines within boxes*), interquartile range (bounds of boxes), and error bars (upper and lower ranges). **c** There was a positive correlation between CD14^++^CD16^+^ expression and the Acute Physiology and Chronic Health Evaluation II (*APACHE II*) score (*n* = 23, Spearman’s correlation coefficient *r* = 0.512, *p* < 0.05). There was also a positive correlation between CD14^++^CD16^+^ expression and the Sequential Organ Failure Assessment (*SOFA*) score (*n* = 23, *r* = 0.439, *p* < 0.05). Linear curves represent least-squares lines. ***p < 0.001

Figure 2a is a representative flow cytometry dot plot of the CD14 and CD16 expression on monocytes cultured for 24 h. Monocytes from sepsis patients showed a significantly higher percentage of CD14^++^CD16^+^ cells than that of healthy volunteers (18.8% vs 7.2%, *p* < 0.001) (Fig. 2b). The percentage of CD14^+^CD16^++^ cells in sepsis patients was somewhat higher than in healthy volunteers, but was statistically insignificant (5.8% vs 3.6%, *p* > 0.05). On the other hand, CD14^++^CD16^–^ cells were significantly lower in the sepsis group (75.0% vs 90.4%, *p* < 0.001) (Fig. 2b). This finding is consistent with previous reports studying CD14^++^CD16^+^ expression on monocytes in sepsis [26].

CD14^++^CD16^+^ monocytes are considered as proinflammatory and characterized by higher expression of proinflammatory cytokines. Cytokine secretion profile in the culture medium of freshly isolated monocytes was compared between sepsis patients and healthy volunteers. Levels of TNF-α, IL-6, and IL-1β in septic patients were significantly higher than those in healthy volunteers (Additional file 1: Figure S1A). The findings were similar to the reports from Munoz et al. [27]. COX-2 protein (Additional file 1: Figure S1B) and its product PGE2 (Additional file 1: Figure S1A) were also significantly increased in monocytes from sepsis patients. The findings are consistent with the report that PGE2 functions to constrain systemic inflammation in sepsis [28].

### Levels of CD14^++^CD16^+^ monocytes correlate with disease severity scores

To highlight the clinical relevance of elevated CD14^++^CD16^+^ monocytes, Spearman’s correlation analysis was performed between levels of CD14^++^CD16^+^ monocytes and disease severity using the APACHE II and the SOFA scores. Positive linear relationships were revealed between levels of CD14^++^CD16^+^ monocytes and APACHE II scores (*r* = 0.512, *p* = 0.013) as well as SOFA scores (*r* = 0.439, *p* = 0.036) (Fig. 2c). However, neither APACHE II score (*r* = –0.377, *p* = 0.101) nor SOFA score (*r* = –0.149, *p* = 0.531) was significantly correlated with CD14^+^CD16^++^ expression (data not shown). The main etiology of this sepsis population was pneumonia. The correlations between severity of illness and CD14^++^CD16^+^ expression still existed when only patients with pneumonia were considered with *p* = 0.047 for APACHE II scores (*r* = 0.538) and *p* = 0.049 for SOFA scores (*r* = 0.515).

### ASCs modify expression of monocyte subsets from sepsis patients

**Fig. 3:**
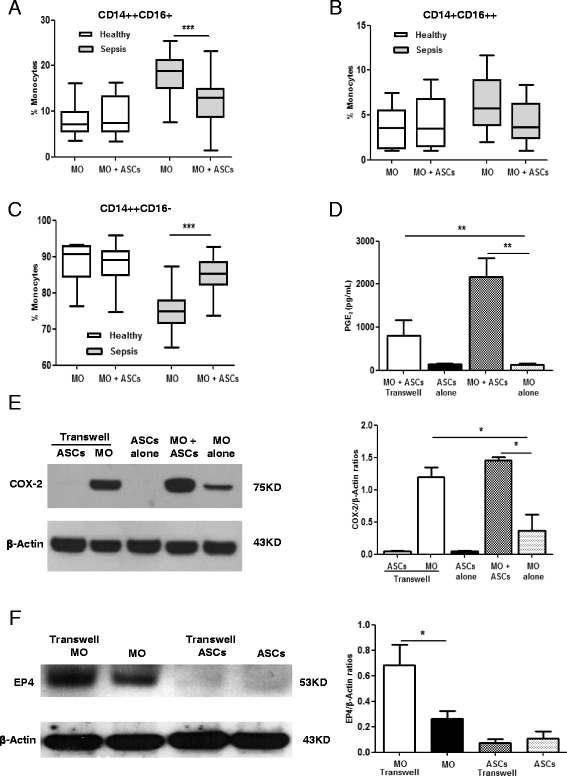
Effect of monocyte-ASC coculture on levels of monocyte subsets, COX-2, PGE2, and EP4. First, monocytes from healthy donors and sepsis patients were cultured alone or cocultured directly with ASCs. The percentage of CD14^++^CD16^+^ (**a**), CD14^+^CD16^++^ (**b**), and CD14^++^CD16^–^ (**c**) monocytes in the total monocyte population were determined via flow cytometry. Box and whisker plots represent median (*lines within boxes*), interquartile range (bounds of boxes), and error bars (upper and lower range); *n* = 25 for healthy donors and *n* = 23 for sepsis patients. ***p < 0.001. Then, ASCs and monocytes from sepsis patients were cultured alone, cocultured directly, or cocultured via Transwell for 24 h. Culture supernatants from the above wells were harvested for quantification of PGE2 via ELISA (**d**). Lysates from different groups were analyzed for COX-2 and EP4 levels via Western blotting. Representative blots and normalized COX-2 levels (**e**) and EP4 (**f**) are shown. β-actin was used as a protein-loading control. Data are expressed as mean ± SEM; *n* = 5 per group. *p < 0.05, **p < 0.01. *ASC* adipose-derived mesenchymal stem (stromal) cell, *COX-2* cyclooxygenase-2, *EP4* prostaglandin E2 receptor 4, *MO* monocytes, *PGE2* prostaglandin E2

To determine the effects of ASCs on monocytes, the distribution of monocyte subsets was examined after direct coculture of ASCs and monocytes. Coculture with ASCs for 24 h significantly reduced the expression of CD14^++^CD16^+^ cells in patients with severe sepsis and septic shock (18.8% vs 13.0%, *p* < 0.001), but not in healthy volunteers (7.2% vs 7.5%, *p* > 0.05) (Fig. 3a). However, the percentages of CD14^+^CD16^++^ cells were unaffected by coculture with ASCs in both sepsis patients (5.8% vs 3.7%, *p* > 0.05) and healthy volunteers (3.6% vs 3.5%, *p* > 0.05) (Fig. 3b). On the other hand, the expression of CD14^++^CD16^–^ monocytes was significantly increased in sepsis patients after coculture with ASCs (75.0% vs 86.0%, *p* < 0.001) (Fig. 3c). These results indicate that ASCs were able to transform proinflammatory CD14^++^CD16^+^ monocytes toward the classic CD14^++^CD16^–^ subsets.

### ASCs modulate levels of COX-2, PGE2, and EP in monocytes

To study the mechanisms involved in the ASC-mediated regulation on monocytes in severe sepsis and septic shock, the expression of COX-2, PGE2, and EP1–4 was examined. To determine the cell source of COX-2 and PGE2, ASCs and monocytes from sepsis patients were cultured alone, cocultured directly, or cocultured via Transwell. The supernatants from the above experiments were analyzed for PGE2 levels. Although both ASCs and monocytes produced very low levels of PGE2 when cultured individually, PGE2 levels were significantly elevated during direct coculture or Transwell (*p* < 0.01) (Fig. 3d). As shown in Fig. 3e, COX-2 expression in ASCs was undetectable during monoculture or coculture via Transwell, indicating that ASCs do not produce detectable COX-2 in the current system. On the other hand, COX-2 was constitutively expressed in monocytes. The COX-2 expression in monocytes was significantly upregulated in direct coculture and Transwell (*p* < 0.05). Therefore, ASCs facilitate the production of COX-2 and PGE2 from monocytes. To study whether ASCs regulate the downstream events of PGE2, the expression profile of four EP receptors was examined in monocytes. Coculture of ASCs with monocytes from sepsis patients significantly increased the expression of EP4 (*p* < 0.05) (Fig. 3f). However, there was no significant change in EP1–3 protein levels (data not shown).

### ASCs affect CD14^++^CD16^+^ expression on monocytes via PGE2 signaling

**Fig. 4:**
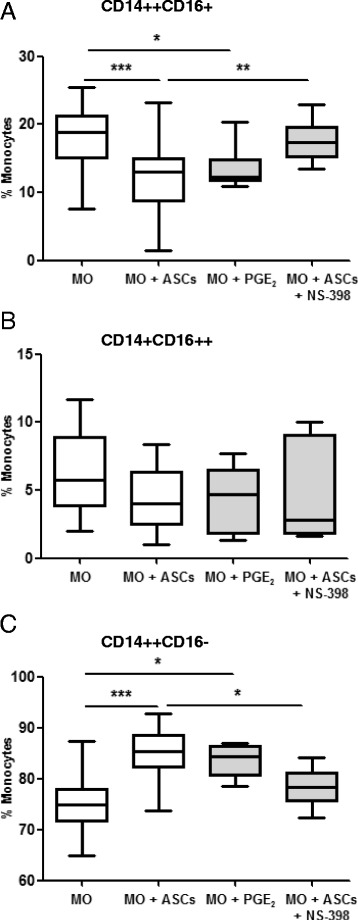
Involvement of PGE2 on the regulation of CD14^++^CD16^+^ monocyte levels. Monocytes from sepsis patients (1 × 10^6^ PBMC/well in six-well plates) were cultured in the presence of ASCs (250,000 cells/well) (*n* = 23) or PGE2 (4 ng/ml, *n* = 9). Separately, monocytes were cultured with ASCs and treated with the PGE2 inhibitor NS-398 (1 μM, *n* = 9). After 24 h, phenotypic analysis was performed to check the three monocyte subsets. The box plot showed the percentage of CD14^++^CD16^+^ monocytes (**a**), CD14^+^CD16^++^ monocytes (**b**), and CD14^++^CD16^–^ monocytes (**c**). The plot shows a median (*lines within boxes*), interquartile range (bounds of boxes), and error bars (upper and lower ranges). *p < 0.05, **p < 0.01, ***p < 0.001. *ASC* adipose-derived mesenchymal stem (stromal) cell, *MO* monocytes, *PGE2* prostaglandin E2

To examine whether PGE2 was responsible for the modulation of monocyte phenotypes, monocytes from sepsis patients were cocultured with ASCs directly or treated with exogenous PGE2. PGE2 mimicked the effect of ASCs in reducing the CD14^++^CD16^+^ phenotype and increasing the CD14^++^CD16^–^ phenotype at 4 ng/ml (Fig. 4a and c). Moreover, the COX-2-specific inhibitor NS-398 at 1 μM abolished the effect of ASCs in regulating the CD14^++^CD16^+^ and CD14^++^CD16^–^ phenotypes (Fig. 4a and c). On the other hand, both NS-398 and PGE2 did not affect the CD14^+^CD16^++^ expression (*p* > 0.05) (Fig. 4b). These data support the notion that PGE2 is directly involved in regulating CD14^++^CD16^+^ expression in monocytes from sepsis patients.

### ASCs modulate monocyte cytokine production via PGE2

**Fig. 5:**
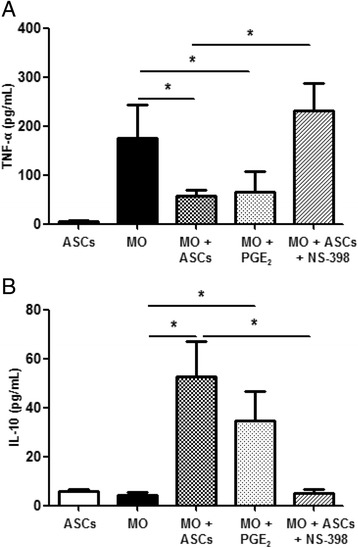
Effect of PGE2 on cytokine levels produced by monocytes from septic patients. Monocytes from septic patients (1 × 10^6^ PBMC/well in six-well plates) and ASCs (250,000 cells/well) were cultured alone or cocultured directly for 24 h. In another group, monocytes were incubated with exogenous PGE2 (4 ng/ml) for 24 h. Separately, monocyte-ASC cocultures were treated with NS-398 (1 μM) for 24 h. TNF-α (**a**) and IL-10 (**b**) levels in the culture supernatant were determined via ELISA. Data are expressed as mean ± SEM; *n* = 7 per group. *p < 0.05. *ASC* adipose-derived mesenchymal stem (stromal) cell, *IL* interleukin, *MO* monocytes, *PGE2* prostaglandin E2, *TNF* tumor necrosis factor

To study the effect of ASCs on monocyte cytokine production, monocytes from sepsis patients were cultured alone or cocultured directly with ASCs and then examined for TNF-α and IL-10 levels in the media. As shown in Fig. 5, ASCs reduced the TNF-α and increased the IL-10 levels in septic monocytes. To test the role of PGE2 in the regulation of the inflammatory cytokines, the monocytes were incubated with exogenous PGE2. Separately, monocytes were cocultured with ASCs and treated with the COX-2-specific inhibitor NS-398. PGE2 mimicked the effect of ASCs in decreasing levels of TNF-α and elevating levels of IL-10 (*p* < 0.05). In contrast, the effects of ASCs on TNF-α and IL-10 were abolished by NS-398 (*p* < 0.05) (Fig. 5). It was been shown that COX-2 was produced by monocytes but not ASCs in coculture (Fig. 3e). These results indicate that NS-398 abolished the effect of ASCs on cytokine production via inhibiting monocyte COX-2.

### ASCs alter the phenotypes of monocytes in a mouse model of sepsis

**Fig. 6:**
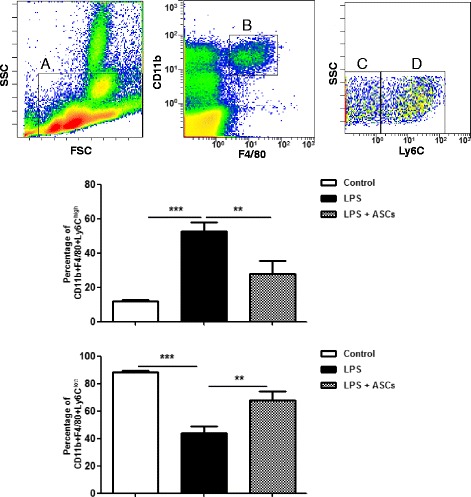
Effect of ASCs on F4/80^+^CD11b^+^Ly6C^high^ monocyte population in vivo. Representative flow cytometry plots of the F4/80^+^CD11b^+^Ly6C^high^ and F4/80^+^CD11b^+^Ly6C^low^ populations from the mouse peripheral blood are shown in the top panel. Monocytes were initially gated by SSC vs FSC (*a*) followed by F4/80 and CD11b (*b*). The F4/80^+^CD11b^+^ cell populations were further gated with Ly6C for F4/80^+^CD11b^+^Ly6C^high^ (*d*) and F4/80^+^CD11b^+^Ly6C^low^ (*c*) cells. The bar graphs summarize the percentage of F4/80^+^CD11b^+^Ly6C^high^ and F4/80^+^CD11b^+^Ly6C^low^ monocytes in the control, LPS, and LPS plus ASC groups. Data represent the mean ± SEM of *n* = 5–6 mice per group. **p < 0.01, ***p < 0.001. *ASC* adipose-derived mesenchymal stem (stromal) cell, *LPS* lipopolysaccharide

Mouse monocytes are classified as proinflammatory F4/80^+^CD11b^+^Ly6C^high^ and patrolling F4/80^+^CD11b^+^Ly6C^low^ monocytes [29]. To determine whether ASCs could modify the distribution of mouse monocytes in vivo, mice were treated with LPS intraperitoneally followed by ASC administration. The distribution of F4/80^+^CD11b^+^Ly6C^high^ and F4/80^+^CD11b^+^Ly6C^low^ monocytes were determined 24 h later via flow cytometry. LPS treatment induced a significant increase in F4/80^+^CD11b^+^Ly6C^high^ and a significant decrease in F4/80^+^CD11b^+^Ly6C^low^ monocytes compared with PBS control (*p* < 0.001). ASC treatment reversed the effect of LPS on monocyte phenotypes in vivo (*p* < 0.01) (Fig. 6).

## Discussion

Both bone marrow-derived MSCs and ASCs have been shown to alleviate sepsis in mouse models [12, 30]. To the best of our knowledge, ours is the first clinical investigation studying the effect of ASCs on monocyte phenotypes in sepsis in vitro. Our results demonstrated that levels of CD14^++^CD16^+^ monocytes in the early phase of severe sepsis and septic shock correlated with APACHE II and SOFA scores. Our study also revealed that ASCs reduced the CD14^++^CD16^+^ phenotype, while increasing levels of CD14^++^CD16^–^ monocytes from sepsis patients. Functionally, ASCs reduced the TNF-α and increased the IL-10 expression in monocytes of septic patients. Mechanistically, monocyte PGE2, COX-2, and EP4 levels increased significantly when ASCs were cocultured with monocytes. Furthermore, exogenous PGE2 mimicked the effect of ASCs on CD14^++^CD16^+^ and cytokine expression. On the contrary, NS-398 abolished the effect of ASCs via inhibiting monocyte COX-2. In addition, ASCs modulated the expression of monocyte phenotypes in a mouse model of sepsis. These results indicate that ASCs may alleviate sepsis via modification of monocyte phenotypes and functions.

To determine the effects of ASCs on monocytes from sepsis patients, we cocultured the two cells types in vitro for 24 h and found that the CD14^++^CD16^+^ phenotype was decreased, while CD14^++^CD16^–^ expression was increased. The phenotypic alteration cannot be attributed to 24 h of in vitro culture. It has been reported that markers for human monocytes remain unchanged until after 2 days of in vitro differentiation [31]. Therefore, ASCs are responsible for the changes in monocyte phenotypes from sepsis patients. Previously, bone marrow-derived MSCs have been reported to skew monocytes towards an antiinflammatory IL-10-producing phenotype [32]. In another study, ASCs induced a phenotypic switch from a proinflammatory macrophage phenotype to an antiinflammatory macrophage phenotype [33].

CD14^++^CD16^+^ monocytes produce higher levels of TNF-α in response to LPS stimulation [34]. CD14^++^CD16^+^ monocytes have been linked to antigen processing and presentation, inflammation, and monocyte activation [35]. There are several reported studies specifically addressing functions of the intermediates. Enhanced frequencies of CD14^++^CD16^+^ but not CD14^+^CD16^++^ have been reported in severe asthmatic patients [36] and glucocorticoid treatment [37]. CD14^++^CD16^+^ monocytes have independently been associated with cardiovascular events in nondialysis patients with chronic kidney disease [38] and in subjects referred for elective coronary angiography [39]. The findings in the present study indicate that CD14^++^CD16^+^ monocytes might serve as a marker for severe sepsis and septic shock.

In the present study, we showed that ASCs regulate the levels of CD14^++^CD16^+^ monocytes from sepsis patients via production of PGE2. Contrary to our expectations, PGE2 was generated from monocytes rather than ASCs. Many believe that paracrine excretion of PGE2 is one of the key mechanisms of MSCs. For example, Nemeth et al. reported that sepsis was alleviated via reprogramming of macrophages by PGE2 from MSCs [12]. However, a multitude of studies simply assume that MSCs are the source of PGE2 when PGE2 is elevated during coculture of MSCs with other cells [40]. The role of PGE2 in regulating monocyte phenotypes has been reported previously. During acute mucosal infection of mice with *Toxoplasma gondii,* monocytes acquire a tissue-specific regulatory phenotype associated with production of PGE2 [41]. PGE2 from monocytes may also be able to potentiate the regulatory effect of MSCs. It has been documented that PGE2 enhances the ability of MSCs to induce regulatory T cells in controlling arteriosclerosis [42]. However, the mechanism leading to the generation of PGE2 in monocytes remains to be solved.

The effects of PGE2 are regulated by the expression pattern of four EP receptor subtypes. Our results showed that ASCs increased EP4 expression in monocytes from sepsis patients. In a sepsis model, Nemeth et al. reported that MSCs ameliorated sepsis and produced PGE2, which targeted on the EP2 and EP4 receptors on macrophages [12]. The PGE2/EP4 pathway was documented as the mechanism for MSC inhibition of T-helper 17 cell differentiation [43]. Recently, Liu et al. reported that MSC-derived microvesicles block the rupture of intracranial aneurysm by suppression of mast cell activation via a PGE2/EP4 mechanism [44].

Our study demonstrates the interaction between ASCs and monocytes. Sepsis is a complex disease which has impacts on both innate and adaptive immunity. During the initial sepsis response, cells of innate immune response such as dendritic cells and monocytes trigger the activation of natural killer (NK) cells along with pathogen-associated molecular patterns to produce inflammatory cytokines. On the other hand, activated T regulatory cells prevent NK cell activation. In the early stage of sepsis, MSCs can exert their effects via interaction with dendritic cells, NK cells, and T regulatory cells. MSCs have been reported to inhibit generation and function of both CD34-derived and monocyte-derived dendritic cells [45]. MSCs can also modulate NK cells by altering the phenotype of NK cells and suppressing proliferation, cytokine secretion, and cytotoxicity against targets [46]. Several studies have shown the capacity of MSCs to promote the generation of T regulatory cells by activating the Notch 1 signaling pathway [47] or through production of HLA-G5 [48].

The present study contributes to understanding the mechanisms of ASCs in sepsis while also leaving a number of unanswered questions that require further exploration. First, it is unclear whether bone marrow-derived MSCs share the same effects as ASCs on monocyte phenotype expression. Second, it is unknown whether ASCs can lead to the direct differentiation of CD14^++^CD16^+^ into CD14^++^CD16^–^ monocytes. Third, the mechanism responsible for the increased production of PGE2 in monocyte-ASC coculture was not fully examined. Further studies are warranted to delineate the mechanisms of ASCs on monocyte phenotypes and functions in septic conditions.

## Conclusions

Levels of proinflammatory CD14^++^CD16^+^ monocytes correlate with APACHE II and SOFA scores in the early phase of severe sepsis and septic shock. ASCs transform monocytes of sepsis patients from CD14^++^CD16^+^ into the CD14^++^CD16^–^ phenotype via a PGE2-dependent mechanism. Further studies in this area may provide new insights into the regulatory mechanisms of ASCs in sepsis and lead to potential clinical applications for ASCs in this condition.

## Additional file

Additional file 1: Figure S1. Cytokines and COX-2/PGE2 levels in freshly isolated monocytes. Monocytes were isolated and cultured for 24 h from sepsis patients (*n* = 6) and healthy volunteers (*n* = 6). Levels of TNF-α, IL-6, IL-1β, and PGE2 (A) in the culture supernatant were determined via ELISA. COX-2 levels in cell lysates were determined via Western blot (B). **p* < 0.05, ***p* < 0.01. (PPTX 161 kb)

## Abbreviations

APACHE: Acute Physiology and Chronic Health Evaluation
ASC: Adipose-derived mesenchymal stem (stromal) cell
COX-2: Cyclooxygenase-2
ELISA: Enzyme-linked immunosorbent assay
EP: Prostaglandin E2 receptor
HGF: Hepatocyte growth factor
ICU: Intensive care unit
IDO: Indoleamine 2,3-dioxygenase
IL: Interleukin
LPS: Lipopolysaccharide
MSC: Mesenchymal stem (stromal) cell
NK: Natural killer
PBMC: Peripheral blood mononuclear cells
PBS: Phosphate-buffered saline
PGE2: Prostaglandin E2
SEM: Standard error of the mean
SOFA: Sequential Organ Failure Assessment
TGF: Transforming growth factor
TNF: Tumor necrosis factor
TSG-6: Tumor necrosis factor-α stimulated gene 6
