# Supplementary figures and images for: Adipose-derived mesenchymal stem cells modulate CD14++CD16+ expression on monocytes from sepsis patients in vitro via prostaglandin E2
Source: Stem Cell Res Ther. 2017 Apr 26;8:97. doi: 10.1186/s13287-017-0546-x (PMC5406890; doi:10.1186/s13287-017-0546-x)

## Slide 1
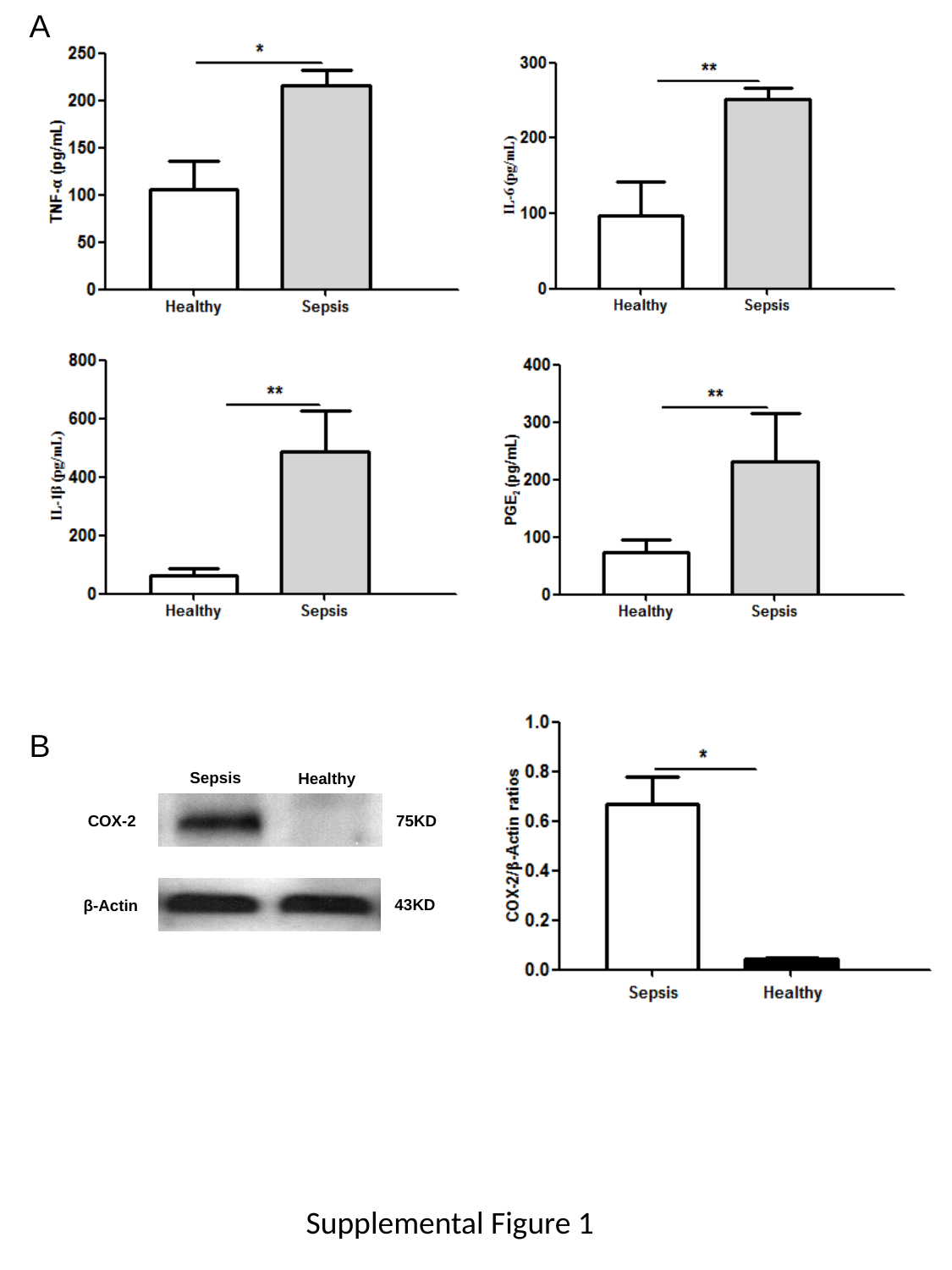

A
B
Sepsis
Healthy
 COX-2
 75KD
 43KD
β-Actin
Supplemental Figure 1

Supplement: Additional file 1: Figure S1. — Cytokines and COX-2/PGE2 levels in freshly isolated monocytes. Monocytes were isolated and cultured for 24 h from sepsis patients (n = 6) and healthy volunteers (n = 6). Levels of TNF-α, IL-6, IL-1β, and PGE2 (A) in the culture supernatant were determined via ELISA. COX-2 levels in cell lysates were determined via Western blot (B). *p < 0.05, **p < 0.01. (PPTX 161 kb) [file 13287_2017_546_MOESM1_ESM.pptx]
